# Supplementary material for: Multi‐dataset identification of innovative feature genes and molecular mechanisms in keratoconus
Source: J Cell Mol Med. 2024 Sep 19;28(18):e70079. doi: 10.1111/jcmm.70079 (PMC11412914; doi:10.1111/jcmm.70079)
Supplement: Supplementary file 2 — Material S2. [file JCMM-28-e70079-s002.zip › 7.GSEA analysis/2.ATOH7/KEGG_GLYCOSAMINOGLYCAN_BIOSYNTHESIS_CHONDROITIN_SULFATE.html]

Details for gene set KEGG\_GLYCOSAMINOGLYCAN\_BIOSYNTHESIS\_CHONDROITIN\_SULFATE[GSEA]

|  || Dataset | ATOH7.group.cls#h\_versus\_l |
| Phenotype | group.cls#h\_versus\_l |
| Upregulated in class | h |
| GeneSet | KEGG\_GLYCOSAMINOGLYCAN\_BIOSYNTHESIS\_CHONDROITIN\_SULFATE |
| Enrichment Score (ES) | 0.6036558 |
| Normalized Enrichment Score (NES) | 1.55554 |
| Nominal p-value | 0.038910504 |
| FDR q-value | 0.25663212 |
| FWER p-Value | 0.694 |
Table: GSEA Results Summary

  

Fig 1: Enrichment plot: KEGG\_GLYCOSAMINOGLYCAN\_BIOSYNTHESIS\_CHONDROITIN\_SULFATE      
 Profile of the Running ES Score & Positions of GeneSet Members on the Rank Ordered List

  

| SYMBOL | TITLE | RANK IN GENE LIST | RANK METRIC SCORE | RUNNING ES | CORE ENRICHMENT || 1 | UST | na | 56 | 0.847 | 0.2492 | Yes |
| 2 | CHST14 | na | 967 | 0.453 | 0.3571 | Yes |
| 3 | CSGALNACT1 | na | 1335 | 0.404 | 0.4662 | Yes |
| 4 | CHST7 | na | 2368 | 0.318 | 0.5305 | Yes |
| 5 | CSGALNACT2 | na | 4424 | 0.221 | 0.5367 | Yes |
| 6 | CHST15 | na | 7072 | 0.141 | 0.5023 | Yes |
| 7 | B3GAT2 | na | 7780 | 0.123 | 0.5184 | Yes |
| 8 | CHPF2 | na | 8401 | 0.110 | 0.5332 | Yes |
| 9 | CHST11 | na | 8609 | 0.106 | 0.5587 | Yes |
| 10 | CHST13 | na | 8697 | 0.104 | 0.5872 | Yes |
| 11 | B4GALT7 | na | 9120 | 0.097 | 0.6037 | Yes |
| 12 | B3GAT1 | na | 11158 | 0.064 | 0.5638 | No |
| 13 | DSE | na | 12418 | 0.047 | 0.5416 | No |
| 14 | CHST12 | na | 12912 | 0.041 | 0.5396 | No |
| 15 | XYLT2 | na | 13122 | 0.039 | 0.5452 | No |
| 16 | B3GAT3 | na | 13394 | 0.036 | 0.5481 | No |
| 17 | CHPF | na | 15846 | 0.014 | 0.4817 | No |
| 18 | CHSY1 | na | 15877 | 0.014 | 0.4849 | No |
| 19 | XYLT1 | na | 16288 | 0.010 | 0.4762 | No |
| 20 | B3GALT6 | na | 16308 | 0.010 | 0.4787 | No |
| 21 | CHST3 | na | 21672 | -0.039 | 0.3358 | No |
| 22 | CHSY3 | na | 27412 | -0.136 | 0.2108 | No |
Table: GSEA details [plain text format]

  

Fig 2: KEGG\_GLYCOSAMINOGLYCAN\_BIOSYNTHESIS\_CHONDROITIN\_SULFATE      
 Blue-Pink O' Gram in the Space of the Analyzed GeneSet

  

Fig 3: KEGG\_GLYCOSAMINOGLYCAN\_BIOSYNTHESIS\_CHONDROITIN\_SULFATE: Random ES distribution      
 Gene set null distribution of ES for **KEGG\_GLYCOSAMINOGLYCAN\_BIOSYNTHESIS\_CHONDROITIN\_SULFATE**
